# NucleoFinder: a statistical approach for the detection of nucleosome positions

**DOI:** 10.1093/bioinformatics/bts719

**Published:** 2013-01-06

**Authors:** Jeremie Becker, Christopher Yau, John M. Hancock, Christopher C. Holmes

**Affiliations:** ^1^Department of Statistics, University of Oxford, Oxford OX1 3TG, ^2^MRC Harwell, Harwell Science and Innovation Campus, Oxfordshire OX11 0RD and ^3^Department of Physiology, Development and Neuroscience, Cambridge CB2 3EG, UK

## Abstract

**Motivation**: The identification of nucleosomes along the chromatin is key to understanding their role in the regulation of gene expression and other DNA-related processes. However, current experimental methods (MNase-ChIP, MNase-Seq) sample nucleosome positions from a cell population and contain biases, making thus the precise identification of individual nucleosomes not straightforward. Recent works have only focused on the first point, where noise reduction approaches have been developed to identify nucleosome positions.

**Results:** In this article, we propose a new approach, termed NucleoFinder, that addresses both the positional heterogeneity across cells and experimental biases by seeking nucleosomes consistently positioned in a cell population and showing a significant enrichment relative to a control sample. Despite the absence of validated dataset, we show that our approach (i) detects fewer false positives than two other nucleosome calling methods and (ii) identifies two important features of the nucleosome organization (the nucleosome spacing downstream of active promoters and the enrichment/depletion of GC/AT dinucleotides at the centre of *in vitro* nucleosomes) with equal or greater ability than the other two methods.

**Availability**: The R code of NucleoFinder, an example datafile and instructions are available for download from https://sites.google.com/site/beckerjeremie/

**Contact**: cholmes@stats.ox.ac.uk

**Supplementary information:** Supplementary data are available at *Bioinformatics* online.

## 1 INTRODUCTION

Eukaryotic genomes exist in highly packaged structures called chromatin. DNA wraps around globular protein cores and appears as ‘beads on a string’ by electron microscopy. The ‘bead’, or nucleosome, is the fundamental structural unit of the chromatin and consists of ∼150 base pairs coiled 1.65 times around a histone octamer core comprising two H2A/H2B and two H3/H4 heterodimers (Luger *et al.*, 1997). The ‘string’, also called the linker, is a DNA stretch ranging from 10 to 80 bp, depending on species, cell type and chromatin region. Nucleosomes can further be folded into higher order organizations to eventually form a chromosome (Felsenfeld and Groudine, 2003). The need for these high level structures arises from a packaging problem: the DNA molecule is long (chromosomes range from 1.7 to 8.5 cm in human) and negatively charged, resulting in electrostatic repulsions that stiffen the polymer and prevent it from fitting inside the nucleus. Owing to the highly basic charge of histone proteins, DNA charges are neutralized, allowing a compaction up to a factor of 10 000 (Jiang and Pugh, 2009).

This compaction is not homogeneous throughout the genome and restricts both the binding of transcription factors and the recruitment of effector protein complexes (such as chromatin remodelling complexes) to certain regions (Millar and Grunstein, 2006). Chromatin has commonly been viewed as being in either an active form (euchromatin) or a repressed form (heterochromatin). The former, characteristic of active transcribed regions, is lightly packed (up to the 30 nm fiber compaction) and allows regulatory proteins and transcription complexes to bind to the DNA. The latter, tightly packed, is associated with gene silencing and the protection of chromosome structures such as centromeres and telomeres (Elgin, 1996). Because of its influence on the binding of transcription factors, the packaging of DNA into nucleosomes provides important regulatory functions (Yuan *et al.*, 2005). In yeast for instance, Shivaswamy *et al.* (2008) demonstrated that gene activation and repression are associated with changes (eviction, appearance or repositioning) of one or two nucleosomes in promoter regions.

The development of high-throughput assays has allowed the mapping of nucleosomes across the genomes of various model organisms (Lee *et al.*, 2007; Mavrich *et al.*, 2008; Schones *et al.*, 2008; Valouev *et al.*, 2008). These recent studies have revealed common features across species : at expressed genes (i) the 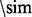
150 base pairs region upstream of the transcription start site (TSS) is depleted of nucleosomes, (ii) nucleosomes show a regular spacing at the 5′ end of genes and (iii) this regular nucleosome spacing, also called phasing, gradually decreases from the 5′ to the 3′ end of the coding region (Bai and Morozov, 2010). Even if our understanding of the nucleosome organization has considerably improved, the questions of the relative influence of *cis* and *trans* factors in the nucleosome organization as well as the role of nucleosome positioning in gene regulation remain active areas of research. A major challenge in addressing these questions is that the precise identification of individual nucleosomes is rendered complex by current assays which (i) are biased and (ii) sample nucleosomes positions across a cell population, as discussed below, raising the need for nucleosome calling methods that account for both issues.

The main experimental bias arises from the use of the micrococcal nuclease (MNase) to isolate the DNA fragments bound to histones (nucleosomal DNA). In addition to digesting naked DNA, the MNase is known to cleave AT/TA dinucleotide motifs as well as showing other, more subtle, sequence specificity (Horz and Altenburger, 1981). After measuring the nucleosomal DNA either by microarray (MNase-chip) or deep sequencing (MNase-seq), the resulting nucleosome map thus reflects regions both depleted of nucleosomes and rich in AT/TA dinucleotides. Even though Kaplan *et al.*, (2010) showed that nucleosome protection is the dominant factor in MNase digestion, it is desirable to control for this bias along other potential experimental biases. In MNase-Chip experiments, this is done by hybridizing nucleosomal DNA against a control sample (naked genomic DNA). By contrast, in MNase-Seq experiments, because nucleosomal and control DNA are assayed separately, the experimental biases can only be corrected at the nucleosome calling stage. However, because the majority of the MNase-Seq studies do not include control samples (notable exceptions including Valouev *et al.*, 2008, 2011; Zhang *et al.*, 2009), current nucleosome calling methods tailored to MNase-Seq data do not take into account control samples.

Regarding the second point, because nucleosomes are assayed in a cell population, their associated reads (or intensities in the case of MNase-Chip) are either normally distributed within ∼15 bp of the nucleosome centre when highly phased, or uniformly distributed otherwise (Jiang and Pugh, 2009). This variation in read positions reflects both the positional heterogeneity across cells and the over-trimming or under-trimming of the DNA at nucleosome borders during sample preparation.

Nucleosome calling methods recently developed aim to identify the footprint of phased nucleosomes from a signal processing perspective. They commonly involve two steps of background noise filtering and peaks scoring. These methods include Laplacian of Gaussian (Zhang *et al.*, 2008a), Fourier Transform (Flores and Orozco, 2011), correlation with nucleosome footprint (Weiner *et al.*, 2010), mixed methods (Di Ges *et al.*, 2009) for MNase-Seq data and Hidden Markov Models for MNase-Chip data (Kuan *et al.*, 2009; Lee *et al.*, 2007; Yassour *et al.*, 2008; Yuan *et al.*, 2005). However, no current methods include control data that can provide important additional information for inferring nucleosome positioning.

In this article, we introduce NucleoFinder, a new statistical approach for MNase-Seq data analysis that aims to identify nucleosomes consistently positioned in a cell population (phased) and showing a significant enrichment relative to a control sample. NucleoFinder is based on nucleosome positioning, a concept commonly used in MNase-Seq analyses (Field *et al.*, 2008; Kaplan *et al.*, 2009; Valouev *et al.*, 2008, 2011; Zhang *et al.*, 2009) to quantify the extent to which nucleosome positions vary across the cell population assayed. This statistic relates the number of read counts in a 30 bp window (to account for the MNase cleavage uncertainty, Jiang and Pugh, 2009) to the number of read counts in the two 60 bp flanking windows. The larger is this ratio, the more precisely positioned the nucleosome is. To take into account the control information in the nucleosome positioning measurement, we extend this idea by looking for the pattern *not enriched*, *enriched*, *not enriched* between the nucleosome and control samples in three consecutive windows 60, 30, 60 bp long.

Our first attempt was based on the binomial test that measures the relative enrichment in the nucleosome sample relatively to the control sample. Because it is based on the relative enrichment, this test did not deal well with background noise and led to a high false positive rate: when in a given region, few random reads (a couple) were present in the nucleosome sample but not in the control sample, the null hypothesis (equal enrichment in both samples) was rejected and the region declared as nucleosome. To circumvent this, we model the difference in read counts between nucleosome and control samples. The difference is truncated to zero, as negative values do not provide any additional information apart from the fact that the considered region is not enriched. This truncated difference is then modelled using a Poisson distribution where prior distributions on the rate parameter are either gamma or uniform. Lastly, to distinguish relatively high background noise from local peaks in the two side windows, we split them into two 30 bp long windows. Ideally, even smaller windows would be preferable to differentiate regions where reads cluster from those with a high number of randomly distributed reads, but the ∼30 bp measurement uncertainty prevents us to do so.

## 2 METHODS

In this section, we provide details on the data and statistical model developed.

### 2.1 Data

The dataset and preprocessing steps used in this analysis are taken from Valouev *et al.*(2011), where nucleosomes were isolated from three haematopoietic cell lines (CD4+, CD8+ T cells and granulocytes) as well as *in vitro* and in a control samples. They were then treated with micrococcal nuclease to release nucleosome cores, and deep sequenced using the SOLiD platform (Pandey *et al.*, 2008). The resulting 25 bp reads derived from the 5′ end of nucleosomal DNA were subsequently mapped against the human hg18 assembly and yielded a genome coverage ranging between 16–28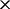. Reads that mapped to more than one single location were discarded. Following the data processing used by Zhang *et al.* (2008b), (i) genomic positions covered by an excessive number of reads were removed (because likely to arise from amplification and sequencing errors, Zhang *et al.*, 2008b), and (ii) nucleosome centres were obtained by adding 75 bp to the 5′ end position of the reads (Zhang *et al.*, 2009). Reads position were then binned into 30 bp windows in each dataset. The CD4+, CD8+ T cells, granulocytes and *in vitro* samples were finally corrected by subtracting the number of reads in each 30 bp window by the number of reads in the control falling in the same window. Negative values were truncated to 0.

### 2.2 Statistical model

#### 2.2.1 Likelihood

Given a 150 bp region, let 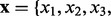

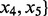
 denote the corrected number of nucleosome centres falling in each of the five 30 bp bins. The bins are grouped into three segments 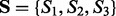 that include bins {{1,2},{3},{4,5}}, respectively. Each random variable 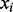 is assumed Poisson distributed with parameter 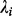. The number of reads is assumed independent from one bin to another, which allows the likelihood to be factorized. The likelihood of 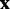 is expressed as:
(1)
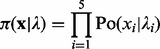



#### 2.2.2 Priors

Whether segment 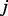 is background or enriched, the prior on 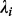 is either:

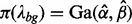
, i.e. gamma distributed with parameters 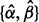 estimated chromosome by chromosome from the data using the method of moments. The hyperparameters are not directly estimated with 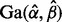 but from the marginal distribution that we introduce below. Since most of the nucleosomes in the human genome show little or no sign of positioning, the data consist mainly of randomly distributed reads. Hence, the estimation of the hyperparameters from the data should allow to capture the background nucleosome distribution.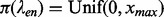, i.e. uniformly distributed in the interval 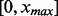, where 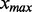 denotes the maximum read counts in the corresponding chromosome 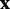. Unlike 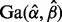 which has most of the mass concentrated around 0, this prior gives same weight to all read counts.

#### 2.2.3 Marginal likelihood

Our main interest is to find regions that match the profile of well-positioned nucleosomes, i.e. when 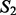 is enriched and 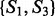 are background. To capture this pattern, we introduce eight models that cover all possible combinations of 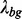 and 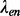 in 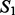, 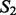 and 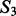, as indicated in Table 1. In each 150 bp sliding region, the marginal likelihoods associated with these eight models are calculated. When 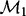 has the largest marginal likelihood among the models, this region is considered as a well-positioned nucleosome. The reason for using eight models is that it allows a better discrimination between well-positioned nucleosomes and background or randomly enriched regions.

**Table 1.** Models Priors: to precisely capture the profile of well-positioned nucleosomes, we introduce eight models that cover all possible combinations of λ*_bg_* and λ*_en_* in *S*_1_, *S*_2_ and *S*_3_

Model	Parameters		
	λ_1,2_ (*S*_1_)	λ_3_(*S*_2_)	λ_4,5_ (*S*_3_)
*M*_0_	λ**_bg_**	λ*_bg_*	λ*_bg_*
*M*_1_	λ*_bg_*	λ*_en_*	λ*_bg_*
*M*_2_	λ*_en_*	λ*_en_*	λ*_en_*
*M*_3_	λ*_en_*	λ*_bg_*	λ*_en_*
*M*_4_	λ*_en_*	λ*_en_*	λ*_bg_*
*M*_5_	λ*_bg_*	λ*_en_*	λ*_en_*
*M*_6_	λ*_en_*	λ*_bg_*	λ*_bg_*
*M*_7_	λ*_bg_*	λ*_bg_*	λ*_en_*

Since the calculation of the marginal likelihood is similar across the models, we only illustrate it with 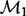, where the priors are as follows:
(2)
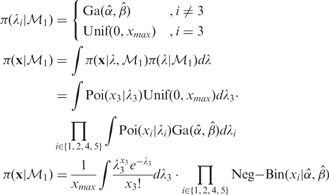

where 
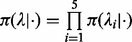


The poisson and gamma distributions being conjugate, the marginal likelihood can be calculated in closed form using the negative-binomial distribution (Gelman *et al.*, 2004). This is, however, not the case with the uniform prior, where the marginal likelihood can nevertheless be calculated by successive integrations by part. To avoid the calculation of this integral every 150 bp window, we compute it for 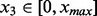 and store the results during the initiation stage of NucleoFinder to speed-up the execution.

As mentioned above, a nucleosome is detected when the marginal likelihood associated with 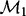 is the largest of all models. In addition to the information of the presence/absence of a nucleosome, it would be desirable to have a sense of how enriched and well-positioned are the detected regions. A good measurement is provided by the Bayes factor between 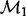 and 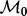, which measures how much the data support 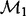 compared with the null model made of background priors only:
(3)
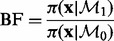



## 3 RESULTS

An important feature of the nucleosome organization in eukaryotic genomes is that nucleosome positions are variable across conditions, tissues and individuals. This implies that large-scale regions consistently occupied by nucleosomes on which nucleosome calling methods could be compared do not exist. Even in yeast, considered mainly populated by well-positioned nucleosomes (Radman-Livaja and Rando, 2010), substantial divergences have been found across studies. An example is provided in Yassour *et al.* (2008) where the authors compared their predictions with a small set of experimentally verified nucleosomes taken in Segal *et al.* (2006). The authors found that neither their predictions nor those of Yuan *et al.* (2005) and Lee *et al.* (2007) are consistent with this reference dataset. This means that there are no true positives available on which nucleosome calling methods can be tested with high confidence.

Despite this absence of large-scale validated dataset, we compare the performance of NucleoFinder with two popular (MNase-Seq) nucleosome calling methods, NPS (Zhang *et al.*, 2008a) and TemplateFilter (Weiner *et al.*, 2010) based on (i) their specificity, (ii) their ability to identify the uniform nucleosome spacing occurring near active promoters *in vivo* and (iii) their ability to detect the stereotypical A/T and G/C dinucleotide patterns present at well-positioned nucleosomes *in vitro* (Valouev *et al.*, 2011). These two methods are run with the parameters set to their default values. To rule out the possibility that the signature detected by these methods could be identified by simple clustering, we also run K-means by iteratively increasing the number 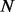 of clusters until one of them matches the pattern of well-positioned nucleosomes (i.e. *not enriched*, *enriched*, *not enriched* in three consecutive windows 60, 30, 60 bp long). In the following analysis, 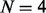.

### 3.1 Specificity

**Fig. 1.**
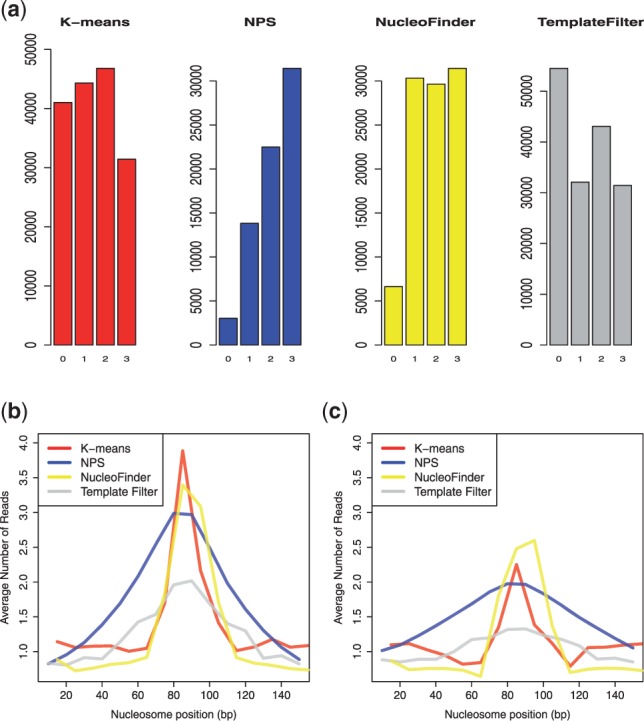
(**a**) For each method, the number of common predictions with 0, … , 3 other methods is represented. (**b**) Nucleosome profile before reads permutation, and after (**c**) in chromsome 22: reads count are averaged in 10 bp bins along nucleosomes predicted by K-means, NPS, NucleoFinder and TemplateFilter

To have a general idea of how NucleoFinder relates to K-means, NPS and TemplateFilter, the four methods are first compared on chromosome 22 (CD4+T cells) where common predictions across methods, defined as the predictions whose central 30 bp overlap, are counted. Out of 247 874 predicted regions, 23.5, 18.8 and 12.7% were common to two, three or four methods, that is, more than half of them are predicted by at least two methods. This result indicates that, although agreeing on a large number of regions, these four approaches capture slightly different aspect of the nucleosome signature. When looking at each method individually, it can be noticed that the total number of predictions (Table 2) as well as the proportion of method-specific predictions is much larger in K-means and TemplateFilter than in NPS and NucleoFinder (Fig. 1a). These large numbers of predictions found with K-means and TemplateFilter suggest that they either have higher sensitivity or perform less well.

**Table 2.** Number of nucleosome predictions in chromosome 22 before and after permutation

Method	Before permutation	After permutation	Specificity (%)
K-means	163 584	203 976	94.20
NPS	70 787	68 099	98.06
NucleoFinder	96 821	27 218	99.22
TemplateFilter	161 116	205 768	94.14

We then estimate the specificity of these four methods by randomly permuting reads in 1 kb windows along chromosome 22. Even though the locations of well-positioned nucleosomes are unknown, the best method is that for which the number of predictions is large before permutation and small after (suggesting both a good sensitivity and specificity). The specificity is calculated by considering the predictions after permutation as false positives (FP, it is not excluded that nucleosome-like patterns occur by chance after permutation though) and the remaining regions (not called) as true negatives (TN):
(4)
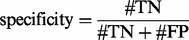



A total of 203 976, 68 099, 27 218 and 205 768 false positives are generated by K-means, NPS, NucleoFinder and TemplateFilter, respectively, resulting in a higher specificity of NPS and NucleoFinder relative to K-means and TemplateFilter (Table 2). If we compare the number of predictions before and after permutation, we observe a 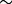
25% increase for K-means and TemplateFilter, a nearly identical result for NPS, and a decrease of 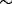
70% for NucleoFinder. The fact that after permutation, NucleoFinder has the smallest number of predictions and the largest decrease in prediction supports the idea that it is specific of well-positioned nucleosomes.

However, the results of K-means after permutation should be interpreted with caution as they rely on the assumption that the data consist of genuine nucleosome signal: after K-means has clustered the data into four groups, the label ‘nucleosome’ is assigned to the cluster (its associated regions) whose profile matches that of nucleosome. This means that whether the input data contain good or poor quality data (permuted data), this approach always generates nucleosome predictions.

The large number of false positive found by TemplateFilter is, on the other hand, probably owing to the variety of templates used by this method to detect nucleosomes. The representative templates, inferred from the authors’ data, are correlated with forward- and reverse-strand reads for various distances between the two strands (the correlation threshold used to call a nucleosome can be adjusted by the user). The important variability across templates (one or two peaks with different widths, see Supplementary Fig. S1, Weiner *et al.*, 2010) suggests that they can capture various nucleosome profiles (high sensitivity), but at the same time, are more likely to pick up nucleosome-like patterns (or background noise) present after permutation. This idea is coherent with the relatively flat nucleosome profiles found with TemplateFilter before and after read permutation (Fig. 1b and c, see explanation below), corresponding to a mixture of templates with different shapes. One possible explanation for which NucleoFinder suffers less from the permuted data is that it explicitly models the null and the alternative hypotheses (background and enriched regions).

To further understand the characteristic of these four methods, a stereotypical profile is built for each of them by averaging the read counts in 10 bp bins along their predictions, before and after permutation. Before permutation, the four methods result in fairly different bell-shaped profiles where unlike TemplateFilter, both K-means and NucleoFinder profiles reveal a sharp enrichment in the central 30 bp window and a low background in the side windows (Fig. 1b). After permutation, the average level of enrichment decreases substantially in all four methods and, except for K-means and NucleoFinder, their profiles flatten out. Although both have a pronounced enrichment in the central segment, these last two methods resulted in very different numbers of predictions (Table 2). This difference is likely owing to the higher stringency of NucleoFinder in the side segments, as the low average read counts shows (Fig. 1b and c). This is consistent with the high specificity of NucleoFinder described earlier and supports the idea that NucleoFinder only detects the signature of well-positioned nucleosomes.

### 3.2 Inter-nucleosome distance comparison

According to the barrier model (Mavrich *et al.*, 2008), the steric hindrance imposed by RNA Pol II induces a compact and regularly spaced nucleosome array within ∼1 kb and progressively dissipates when moving away from the TSS. The spacing just downstream of the TSS is therefore expected to be of the same order of magnitude as estimated at active promoters (178–187 bp, Valouev *et al.*, 2011). For each approach, we estimate the inter-nucleosome distance in the 1 kb region downstream of TSSs of highly expressed genes, defined as those with RPKM (reads per kilobase of exon per million mapped reads, Mortazavi *et al.*, 2008) >10. This criterion follows the classification in four gene groups used in Valouev *et al.* (2011). The gene expression data used here come from the same CD4+T cells as those used to assay the nucleosomes (Valouev *et al.*, 2011), available on GEO, accession number GSE25133. To account for missing predictions, nucleosomes are enumerated from the TSS to the 3′ end of the 1 kb window, without constraining them to be consecutive, and a linear model is used to find optimal numbering.

From the inter-nucleosome distances estimated at 2754 highly expressed genes, the mean and standard deviation are computed for each method (Table 3). Substantial differences are found across methods where, taking Valouev *et al.* (2011) value of nucleosome spacing as a reference (182 bp), K-means and TemplateFilter result in an under-estimation of ∼20 bp and ∼10 bp, respectively, unlike NPS and NucleoFinder that provide improved estimations. In addition to being biased, K-means results in a standard deviation almost twice as large as those found in the other methods, indicating that this method perform poorly both in term of accuracy and precision.

**Table 3.** Estimation of the inter-nucleosome distance (mean and standard deviation)

Region/Method	Inter-nucleosome distance	
	Mean	SD
Active promoters	178–187	–
K-means	159	36
NPS	187	21
NucleoFinder	180	24
TemplateFilter	172	20

### 3.3 Ability to recover nucleosome-specific dinucleotides

The relationship between nucleosome position and DNA sequence has been the subject of intense research (Field *et al.*, 2008; Peckham *et al.*, 2007; Tillo *et al.*, 2010; Valouev *et al.*, 2011; Yuan and Liu, 2008), which has revealed that, owing to their poor distortion properties, poly-dA/dT sequences result in nucleosome exclusion, whereas sequences with 10 bp periodicity of AA/TT/TA dinucleotides facilitate the sharp bending of DNA around the nucleosome (Anselmi *et al.*, 1999). In human, Valouev *et al.* (2011) demonstrated that *in vitro*, the centre of well-positioned nucleosomes is characterized by an enrichment of G/C motifs and a depletion of A/T motifs.

This information can be used to compare methods, where a large discrepancy between GC and AT frequencies at the centre of nucleosome predictions *in vitro* will suggest a high specificity of the considered method for well-positioned nucleosomes. To quantify this pattern, we measure the difference between the GC and AA/TT frequencies at the centre of nucleosomes predicted in chromosome 22. To account for the two-fold symmetry axis of the nucleosome structure (Richmond and Davey, 2003), the GC and AA/TT frequencies are calculated from both the sense and antisense sequences associated with the predictions. 95% confidence intervals are then computed for the difference 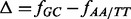:
(5)
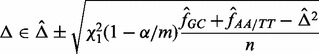

where 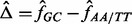, 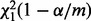 is the 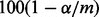
th percentile of the chi-square distribution with 1 degree of freedom, 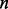 the number of sequences used for the estimation of 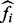 and 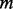 the number of dinucleotides (Goodman, 1965).

**Fig. 2.**
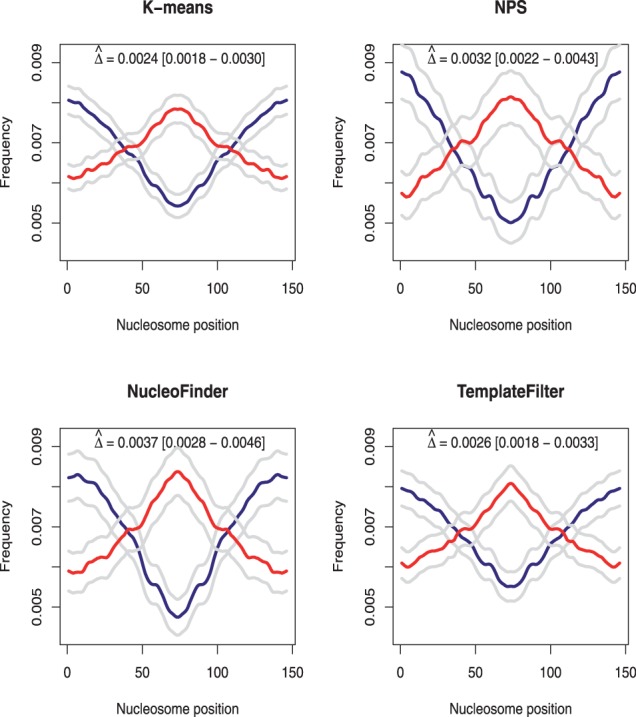
AA/TT (blue) and GC (red) dinucleotide frequencies and 95% confidence intervals along nucleosome predictions in K-means, NPS, NucleoFinder and TemplateFilter. Larger values of 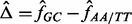 are found in NucleoFinder and NPS than K-means and TemplateFilter

Consistently with their role in promoting or repelling nucleosome, AA/TT and GC frequencies display U-like and bell-like shapes in the four methods (Fig. 2). This pattern is more or less pronounced across methods, as reflected by the values of 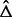 that vary from 0.0024 (0.0018–0.0030) for K-means to 0.0037 (0.0028–0.0046) for NucleoFinder. When looking at the confidence intervals associated with the four methods, it can be noticed that they all overlap (Fig. 2), implying that none of these methods significantly outperform the others. However, the larger values found in NPS and NucleoFinder support the idea that these two approaches have a greater ability to detect well-positioned nucleosomes.

## 4 DISCUSSION

The recent development of high-throughput sequencing methods has provided a global insight into how the combination of nucleosome positions and their post-transcriptional modifications are key to gene regulation. The precise identification of nucleosomes is therefore crucial to understanding what determines the nucleosome architecture and how changes in this organization induce variation in gene expression. This task is made non-trivial by the fact that nucleosomes are assayed across cell populations and consequently result in complex patterns of reads. Another difficulty also arises from the known bias of MNase toward AT/TA motifs, which potentially can limit the accuracy of nucleosome detection. From our knowledge, no existing MNase-Seq methods have addressed this issue. To fill this gap, we introduce NucleoFinder, which looks for regions having both a high level of nucleosome positioning and a significant nucleosome enrichment relative to a control sample.

Despite the absence of large scale regions consistently populated by nucleosomes that could be used for methods comparison, we demonstrated that NucleoFinder is robust to outliers and generates fewer false positives than the other methods. We further showed that NucleoFinder captures important features of the nucleosome organization to an equal or greater extent than the other methods: (i) the estimation of nucleosome spacing from its *in vivo* predictions was consistent with Valouev *et al.* (2011), and (ii) the characteristic G/C-rich and A/T-depleted signals at the centre of *in vitro* nucleosomes was more pronounced at NucleoFinder predictions.

*Funding*: UK Medical Research Council Doctoral Studentship (to J.B.); UK Medical Research Council Specialist Training Fellowship in Biomedical Informatics (Ref No. G0701810 to C.Y.); MRC Programme Leaders Award (to C.C.H.).

*Conflict of Interest*: none declared.
